# Tobacco Taxes as the Unsung Hero: Impact of a Tax Increase on Advancing Sustainable Development in Colombia

**DOI:** 10.3389/ijph.2022.1604353

**Published:** 2022-03-30

**Authors:** Norman Maldonado, Blanca Llorente, Luz Myriam Reynales-Shigematsu, Belen Saenz-de-Miera, Prabhat Jha, Geordan Shannon

**Affiliations:** ^1^ Research Center on Health Economics and Social Protection (PROESA), Universidad ICESI, Cali, Colombia; ^2^ Global Tobacco Economics Consortium (GTEC), Instituto Nacional de Salud Pública, Cuernavaca, Mexico; ^3^ Fundación Anáas, Bogotá, Colombia; ^4^ Instituto Nacional de Salud Pública, Cuernavaca, Mexico; ^5^ Universidad Autónoma de Baja California Sur, La Paz, México; ^6^ Centre for Global Health Research, University of Toronto, Toronto, ON, Canada; ^7^ Institute for Global Health, University College London, London, United Kingdom

**Keywords:** sustainable development, microsimulation, tobacco taxes, artificial societies, individual heterogeneity, tobacco and equity, NCDs prevention

## Abstract

**Objective:** Tobacco taxes are a well-established cost-effective policy to prevent Noncommunicable Diseases. This paper evaluates the expected effects of a tobacco tax increase on the Sustainable Development Goals in Colombia.

**Methods:** We use microsimulation to build an artificial society that mimics the observed characteristics of Colombia’s population, and from there we simulate the behavioral response to a tax increase of COP$4,750 (an increase that has been discussed by policy makers and legislators) and the subsequent effects in all SDGs.

**Results:** The tobacco tax hike reduces the number of smokers (from 4.51 to 3.45 MM smokers) and smoking intensity, resulting in a drop in the number of cigarettes smoked in Colombia (from 332.3 to 215.5 MM of 20-stick packs). Such reduction is expected to decrease premature mortality, healthcare costs, poverty and people facing catastrophic expenditure on healthcare, to increase health, income and gender equity, and to strengthen domestic resource mobilization even in the presence of illicit cigarettes.

**Conclusion:** Tobacco taxes are an effective intervention for public health and a powerful instrument to advance on the 2030 Sustainable Development Agenda.

**Relevance:** A comprehensive analysis of the impact of tobacco taxes on all areas of Sustainable Development is missing in the empirical literature. Such perspective is needed to break the barriers for further tobacco tax increases by gathering wider societal support, especially from stakeholders and key decision makers from development areas other than health.

**SDG Nr:** SDG3 (health), SDG 1 (no poverty), SDG 4 (education), SDG 5 (gender equality), SDG6 (water), SDG10 (inequality), SDG12 (responsible production and consumption), SDG17 (partnerships).

## Introduction

This Original Article is part of the IJPH Special Issue “Health in all Sustainable Development Goals”

Noncommunicable diseases are the leading cause of death and burden of disease worldwide [1]. At the same time, tobacco is one of the greatest risk factors for mortality and morbidity in general, and for NCDs in particular. The effects of tobacco consumption on health and healthcare have been widely estimated, as well as the health effects of cost-effective policies such as tobacco taxes [2, 3]. However, the tobacco epidemic and tobacco control policies also affect other dimensions of Sustainable Development (SD), and those effects have not been comprehensively evaluated. Furthermore, analysis of crucial dimensions of SD such as equity needs to consider social types [4], which calls for methodologies that can accommodate and carefully keep track of the wide heterogeneity of the population, because such heterogeneity ultimately defines the stratifiers connecting structural and intermediate determinants of health [5].

The purpose of this paper is to evaluate the effect of higher tobacco taxes in advancing Sustainable Development in Colombia. To do so, the paper uses microsimulation, a method flexible enough to accommodate the heterogeneity embedded in the multiple dimensions of SD and the specific mechanisms driving gender inequalities. This paper contributes to the literature in framing the discussion on tobacco taxes around the wider concept of SD, overcoming the limitations of the traditional emphasis of tobacco taxes on health and tax revenues. Gender is particularly relevant for research on tobacco taxes and SD because of the role it has in the tobacco epidemic [6, 7], in SD (SGD 5) and in taxation [8]. Colombia is a relevant country because it is a middle income country in Latin America, a region where smoking prevalence is low and where smoking differences by gender are not as profound as they are in other regions (e.g., Asia), obscuring the role of gender in the tobacco epidemic. The paper also illustrates the advantages of using a complex systems’ methodology (microsimulation) to evaluate health policies, capturing the complex connections between health policies, health and the multiple dimensions of SD. Microsimulation has been used in other areas of development as well as in health [9], and there is some work in microsimulation on tobacco taxes [10–14]; however, most of that work is limited to the health dimension of SD with an emphasis on burden of disease, and the work connecting health with SD is considerably scarce.

## Methods

The core concept is Sustainable Development (SD). Theoretically, development has been defined as the study of the enhancement of people’s living conditions [15]; in practice, United Nations has defined SD as a “*multidimensional undertaking to achieve a higher quality of life for all people*” [16], recognizing that such achievement is only possible under a balanced and integrated progress on the economic, the social and the environmental dimensions [17]. As pointed out by [18] such definition holistically intends to “*make sense of the interactions of three complex systems that sustain human life: the world economy, the global society, and the Earth’s physical environment.*” Globally, SD translates into a set of goals intended to improve people’s quality of life and a rich set of targets by 2030 known as the 2030 Agenda [17]. Based on the concept of SD and the multiple and complex connections among all dimensions of SD, this paper evaluates the SD effects of tobacco taxes in Colombia.

Studying an increase in tobacco taxes is relevant for Colombia because, despite the tax hike of this component in 2016–2018 from 700 Colombian Pesos (COP$) to COP$ 2,100, cigarettes are still very cheap in the country [19]. The paper analyzes a tax hike in the specific component of the excise tax from COP$2,350 to COP$7,000. The magnitude of the increase is supported by evidence-based recommendations in tobacco taxes [20, 21], and such magnitude has been the point of reference in policy discussions in the country [22, 23], making the tax change considered in the analysis relevant for policymakers. In addition, tripling the tax has been the policy recommendation in the context of SDGs [24], which is the focus of the paper.

The core method to develop such evaluation is microsimulation, “*a methodology that simulates the states and behaviors of different units —individuals, households, firms, etc.,— as they evolve in a given environment*.” [25]. The artificial society and the static microsimulation was built in three consecutive steps: 1) construction of the synthetic dataset, 2) simulation of behaviors and states, and 3) estimation of aggregate indicators from the simulated microdata. All three steps of the microsimulation were coded and executed in Stata 16.0, and are explained in the following sections. The model is Open Access: all files are available upon request and it is publicly available in (https://github.com/normanmva/MicrosimulationTobaccoTaxSdg).

### Synthetic Dataset

The core dataset used to create the artificial society (the synthetic dataset) was the 2019 Quality of Life Survey (Encuesta de Calidad de Vida–ECV), a yearly nationally representative survey collected by the National Department of Statistics (DANE), that records information on several aspects and dimensions of households’ welfare and life conditions [26]. The survey is accurate for the analysis because 1) it is the one officially used to monitor smoking prevalence, 2) it is designed to measure development, 3) it has microdata at the individual and at the household level, allowing to incorporate individual and household transmission mechanisms of the tobacco tax policy. ECV has 289,558 individuals in 93,993 households and so those are the dimensions of the artificial society represented in the synthetic dataset. By exploiting ECV’s nationally representative design, the expansion factors are used in the synthetic dataset to infer results for Colombia’s population in 2019, that is, 49,670,800 people belonging to 15,999,298 households. The synthetic dataset at the individual level has a unique identifier for every individual, which is defined as the combination of the ECV’s unique identifiers for the house, the household and the member of the household. The core variables for the microsimulation are whether the person smokes and the number of cigarettes smoked per day. The first one is available in the survey for every person aged 10 or older, while the second one is calculated by combining smoking frequency (daily, several times in a week, and less than once in a week) and intensity (number of cigarettes per day), both collected for all smokers. The ECV’s questionnaire uses a standard question from the Global Adult Tobacco Survey to identify current smokers.

Since ECV is a household survey, questions are asked to each respondent in presence of other household members, causing a significant underreport due to social desirability bias [27]; to correct for this bias, we adjust the data on whether the person smokes or not using smoking prevalence by age and gender from the Survey on Consumption of Psychoactive Substances (SCPS) in 2019 [28]. Since the SCPS is designed to measure legal and illegal psychoactive substances consumption, it also uses a standard questionnaire and a protocol designed by the Inter-American Uniform Drug Use Data System to ensure confidentiality and reduce self-report bias [29]. Specifically, the number of smokers in ECV was adjusted by the ratio of SCPS smoking prevalence to ECV smoking prevalence by age group and gender at the population level, using the expansion factors in both surveys. The set of ratios used is shown in Table 1. Once the aggregate number of smokers for each partition (combination of age group and gender) was adjusted by these ratios, the missing smokers were randomly allocated between the nonsmokers in each partition, drawing random numbers from a uniform distribution. This process was repeated 500 times using Monte Carlo simulation [30] to avoid results being dependent on a particular allocation of missing smokers. The smoking frequency and intensity for the missing smokers was allocated as the median value of each variable in the ECV’s observed smokers.

**TABLE 1:** Ratios for correction of social desirability bias (Colombia, 2019).

Age group	Men	Women
[10, 16]	2.75	4.46
[17, 21]	1.56	4.73
[22, 26]	1.31	3.23
[27, 31]	1.41	2.91
[32, 35]	1.22	2.51
[36, 49]	1.14	2.01
[50, 64]	1.07	1.69

As a survey on quality of life, ECV has many variables capturing the main aspects of development at the individual and at the household level. In addition to the demographic variables of age and gender and the smoking variables mentioned before, we also included location, role in the household, education (years of education and highest education level achieved) and income. For location, ECV reports the state (departamento) for each observation, as well as the region (set of departamentos) and whether the household lives in an urban (cabecera) or a rural (centro poblado) area. For income, the ECV has the additional advantage of being the survey officially used to estimate the poverty rate in Colombia. For that reason, the survey records all the income variables needed to estimate households’ total income; in particular, household income is officially defined by DANE as the sum of 1) labor income, 2) capital income, 3) transfers (subsidies), 4) income from sales of some goods (vehicles, real state, home appliances) and 5) imputation of income from household’s housing [31].

### Simulation of Behaviors and States

**FIGURE 1:**
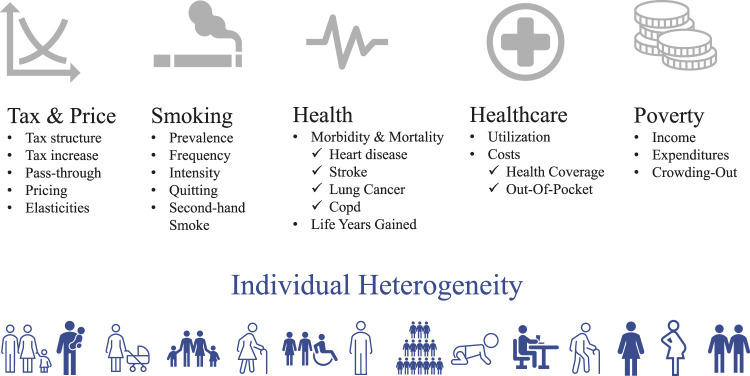
Building blocks of the microsimulation model (Colombia, 2019).

The simulation modeling of behaviors and states builds upon the Extended Cost-Effectiveness Analysis (ECEA) model [32, 33], specifically the aggregated compartmental model used for studying the effect of cigarette price increases in middle-income countries [34]. The ECEA is a static model that analyzes decreases in life expectancy caused by smoking in a cohort of individuals, and the corresponding gains in lives and years of life from increasing tobacco taxes. Therefore, despite being static, the model has the life expectancy of the cohort as an implicit time horizon. The microsimulation structure of the model inherits the structure, time horizon and general properties of the ECEA. It uses the aggregate behavioral and epidemiological parameters to randomly allocate, at the individual level, smoking decisions in response to tax increases and the subsequent health outcomes and use of healthcare. The effects on other dimensions of SD follow either from the SD characteristics of individuals in the synthetic dataset or from additional modeling. We ran 500 Monte Carlo simulations [30] to obtain the probability of different outcomes caused by random allocation of individual behavior. The two alternative scenarios used 100 Monte Carlo simulations. The model’s unit of observation is a person *i* who belongs to the household *h*. The model’s building blocks are summarized in Figure 1 and explained in Supplementary File SC.

### Aggregate Indicators

Finally, the estimation of aggregate indicators is done by defining a metric for the dimensions of SD relevant for tobacco control [35] based on the variables and individual heterogeneity included in the model. We further focus on the SD dimensions mostly related to the household environment because the synthetic dataset is based on a household survey. For the global society, we include health and well-being (SDG3), poverty (SDG1), income equity (SDG10) and gender equality (SDG5). For the world’s economy, we include education (SDG4) and domestic resource mobilization (SDG17). For the earth’s physical environment, we include responsible consumption and production (SDG12) and clean water and sanitation (SDG6). For the sake of precision, when presenting the results, we pinpoint the outcome and the specific SDG Target (SDG-T) or indicator (SDG-I) linked to it.

## Results

A summary of the results is presented in Table 2, and the ones for sensitivity analysis are presented in Table 3. Estimates before and after the tax increase correspond to the average of the estimates in the Monte-Carlo simulations, and the confidence intervals correspond to the 5th and 95th percentiles of the distribution in the Monte-Carlo simulations.

**TABLE 2:** Summary of effects on SDGs from a tax increase that doubles pack prices (Colombia, 2019).

			Before tax increase (baseline)		After tax increase	
System	SDG	Units	Estimate	Conf. Interval	Estimate	Conf. Interval
**Society**	Health (SDG 3)					
	Tobacco tax (specific component) (SDG 3a)	Thousand COP$ per 20-stick pack	2,350.0000	[2,350.0000, 2,350.0000]	7,000.0000	[7,000.0000, 7,000.0000]
	Price	COP$ per 20-stick pack	7,028.6000	[7,028.6000, 7,028.6000]	13,950.3000	[13,950.3000, 13,950.3000]
	# Smokers	Million smokers	4.5073	[4.5069, 4.5122]	3.4485	[3.433, 3.4670]
	Smoking intensity	Cigarettes per day	2.0000	[2.0000, 2.0000]	1.7770	[1.7770, 1.7770]
	Cigarette consumption	Million 20-stick packs	332.4277	[332.2955, 332.5188]	215.5461	[212.3045, 218.8151]
	Deaths (excluding SHS)	Millions	2.2547	[2.2529, 2.2559]	1.8093	[1.789, 1.829]
	Averted deaths from smoking	Thousands			445.3339	[426.0748, 466.3759]
	SHS averted deaths	Thousands			15.9333	[11.2864, 20.6529]
	Healthcare expenditure					
	Healthcare costs savings	MMM COP$			2,158.8017	[1,957.1571, 2,356.076]
	Heart disease	MMM COP$			799.1106	[747.1224, 848.3346]
	Stroke	MMM COP$			693.55480	[635.1308, 754.8049]
	COPD	MMM COP$			344.0871	[307.2251, 381.3045]
	Lung cancer	MMM COP$			322.0492	[267.6787, 371.6319]
	Out of Pocket savings	MMM COP$			194.2922	[176.1441, 212.0468]
	Poverty (SDG 1)					
	Averted poverty	Thousand people			28.9008	[14.7922, 47.9571]
	Averted catastrophic expenditure	Thousand people			337.6163	[296.3577, 379.7444]
	Gender (SDG 5)					
	# Smokers					
	Men	Million	2.8455	[2.8443, 2.8481]	2.1773	[2.1556, 2.1977]
	Women	Million	1.6618	[1.6611, 1.665]	1.2713	[1.2542, 1.2915]
	SHS averted deaths					
	Men	Thousands			7.2059	[4.1213, 11.2842]
	Women	Thousands			8.7152	[5.1394, 13.2422]
**Economy**	Education (SDG 4)					
	Averted loss of knowledge capital per smoker	Years of education			10.1180	[9, 11]
	Averted loss of knowledge capital (total)	Million years of education			3.3685	[3.1504, 3.5999]
	Domestic resources (SDG 17)					
	Tobacco tax revenue (Specific component)	MMM COP$	797.4834	[797.1662, 797.6947]	1,359.8950	[1,337.1956, 1,382.7563]
	Tobacco tax revenue (Specific component) adjusted for smuggled surplus	MMM COP$	1,089.6746	[1,089.6746, 1,089.6746]	2,157.9202	[2,135.0359, 2,180.6037]
**Earth**	Reduction in cigarette butts littered (SDG 12)	Million cigarette butts			1,753.2242	[1,704.6168, 1802.2621]
	Water pollution avoided (SDG 6)	Thousand Million liters			1,753.2242	[1,704.6168, 1802.2621]

**TABLE 3:** Sensitivity analysis (Colombia, 2019).

System	SDG	Units	Before tax increase	After tax increase		
**Society**	Health (SDG 3)					
	Tobacco tax (specific component) (SDG 3a)	Thousand COP$ per 20-stick pack	2,350.00	7,000.00	4,200.00	2,600.00
	Price	COP$ per 20-stick pack	7,028.60	13,950.30	9,582.35	7,086.35
	Number of smokers	Million smokers	4.51	3.45	4.12	4.50
	Smoking intensity	Cigarettes per day	2.00	1.78	1.90	2.00
	Total cigarette consumption	Million 20-stick packs	332.27	215.45	285.80	331.50
	Tobacco-attributable deaths (excluding SHS)	Millions	2.25	1.80	2.10	2.30
	Averted deaths from smoking	Thousands		445.02	165.70	3.60
	Second Hand Smoke (SHS) averted deaths	Thousands		15.94	5.82	0.20
	Healthcare expenditure					
	Healthcare costs savings	MMM COP$		2,157.43	800.90	17.00
	Heart disease	MMM COP$		798.74	299.50	6.69
	Stroke	MMM COP$		692.87	256.80	5.00
	COPD	MMM COP$		343.72	126.80	2.12
	Lung cancer	MMM COP$		322.10	117.80	3.19
	Out of Pocket savings	MMM COP$		194.17	72.10	1.50
	Poverty (SDG 1)					
	Averted poverty cases	Thousand people		28.96	10.90	0.63
	Averted catastrophic expenditure cases	Thousand people		337.60	126.70	2.82
	Gender (SDG 5)					
	Number of smokers					
	Men	Million	2.85	2.18	2.60	2.84
	Women	Million	1.66	1.27	1.52	1.66
	Second Hand Smoke (SHS) averted deaths					
	Men	Thousands		8.09	2.55	0.05
	Women	Thousands		8.26	3.27	0.06
**Economy**	Education (SDG 4)					
	Averted loss of knowledge capital per smoker	Years of education		10.12	9.22	8.56
	Averted loss of knowledge capital (total)	Million years of education		3.37	1.25	0.03
	Domestic resources (SDG 17)					
	Tobacco tax revenue (Specific component)	MMM COP$	797.10	1,359.30	1,111.20	807.00
	Tobacco tax revenue (Specific component) adjusted for smuggled surplus	MMM COP$	1,089.70	2,158.30	1,590.00	1,089.70
**Earth**	Reduction in cigarette butts littered (SDG 12)	Million cigarette butts		1,752.30	697.05	11.55
	Water pollution avoided (SDG 6)	Thousand Million liters		1,752.30	697.05	11.55

### System 1: The Society

#### Health (SDG3)

The tax increase represents an important progress on implementation of the Framework Convention on Tobacco Control (SDG-T 3.a) [36], specifically implementation of tax measures to reduce the demand for tobacco (Article 6). The tax hike in the specific component from COP$ 2,350 to COP$7,000 increases the retail price of a 20-stick pack in 98.4% from COP$7,028.6 to COP$ 13,950.3. Based on data on exchange rates from the Central Bank of Colombia and the Worldbank (annual average of the monthly exchange rate for 2019 of COP$ 3,281.3 per US dollar–US$ and PPP conversion factor for Colombia in 2019 of COP$ 1,317.1 per international $–INT$), the price change equals to an increase from US$ 2.14/INT$ 5.33 to US$ 4.25/INT$10.59.

**FIGURE 2:**
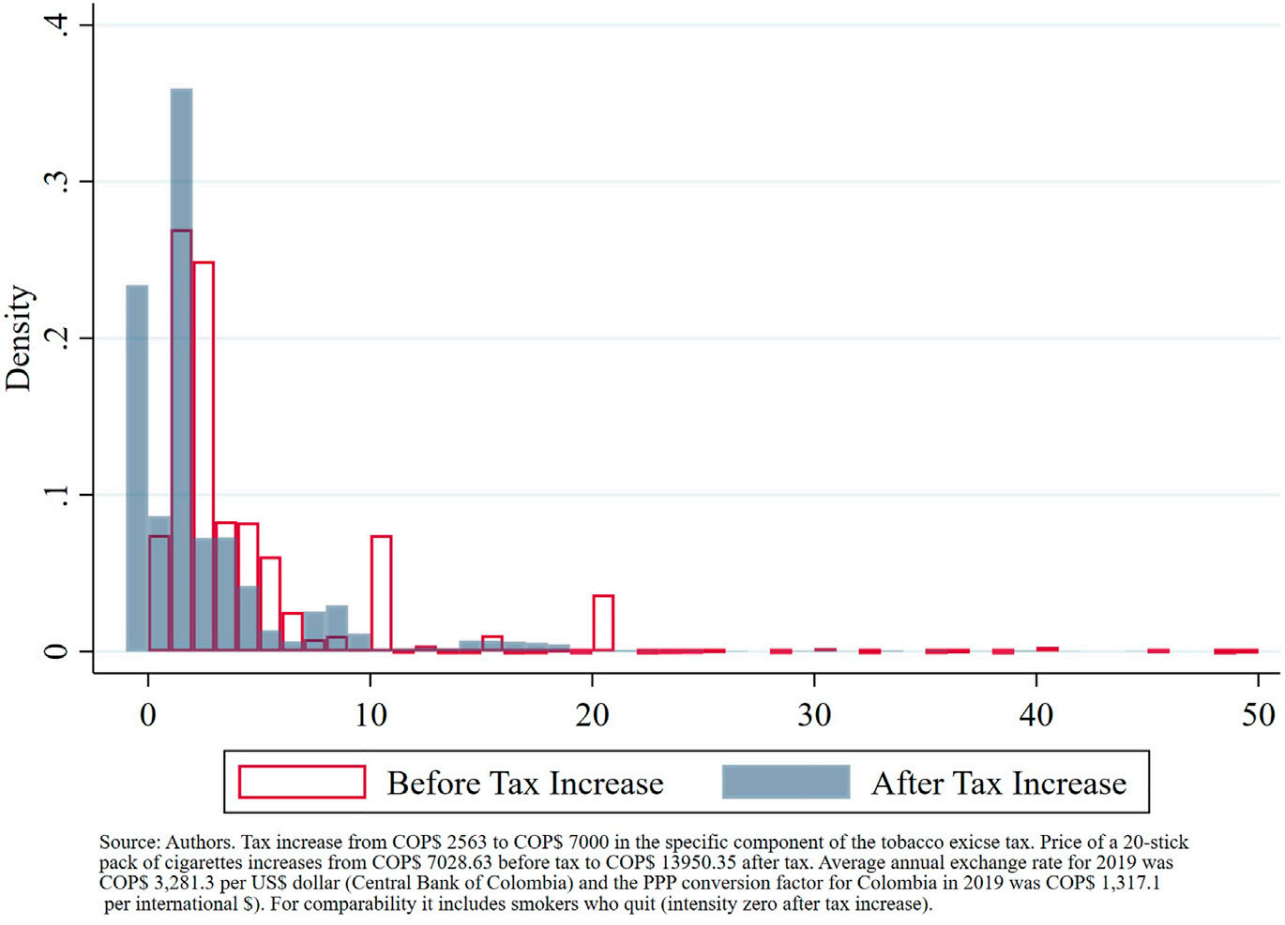
Distribution of smoking intensity in cigarettes per day (Colombia, 2019).

Reduction in demand for cigarettes translates into lower exposure to tobacco, one of the main risk factors of Non-Communicable Diseases (NCDs). At the extensive margin, the tax increase is estimated to reduce the number of smokers from 4.51 to 3.45 million smokers. At the intensive margin, smokers who keep smoking after the tax increase also reduced their consumption, lowering their exposition to the risk factor from 2.01 to 1.78 cigarettes per day (median). Out of both margins, only the reduction in the number of smokers leads to gains in health; in other words, reduction in smoking intensity from non-quitters does not lead to gains in health as the health benefits of smoking reduction, specially if it is not substantial, are not significant [37]. The combination of the effect on smoking at both the extensive and intensive margins is shown in Figure 2. Both effects combined reduce the number of annual cigarettes smoked in Colombia from 332.3 to 215.5 MM of 20-stick packs.

The decrease in the number of smokers reduces the health risk on NCDs, decreasing the premature mortality caused by smoking from 2.3 to 1.8 MM deaths, representing a progress of 445.0 M averted deaths (SDG-T3.4): 233.2 M from heart disease, 99.6 M from stroke, 82.3 M from COPD and 30.0 M from lung cancer. As of July 15, 2021 the number of deaths due to Covid-19 in Colombia was 113,839; also, in 2019, Colombia had 244,355 non-fetal deaths; finally, the armed conflict between 1958 and 2012 in Colombia caused at least 220,000 deaths [38]. Therefore, the tobacco tax hike averts the equivalent of 3.91 times the total deaths from Covid-19, 1.82 times the number of annual non-fetal deaths in Colombia, and 2.02 times the deaths from the armed conflict. The averted deaths translate into a total gain of 7.9 million years of life. In addition to these gains, the tax also averts 15.9 M deaths from exposition to second-hand smoke (SHS) in the household (at home).

#### Healthcare Expenditure

The averted morbidity and mortality from both smoking and second-hand smoking has direct effects on health systems as it translates in lower healthcare utilization and thus, to lower costs. The estimates suggest that the tax increase reduces healthcare costs by COP$2,157.4 MMM: COP$798,740 MM from heart disease, COP$692,868.8 MM from stroke, COP$343,721.3 MM from COPD and COP$322,095.1 MM from cancer. Healthcare for NCDs is expensive and, when a significant part of those costs are covered by Out-Of-Pocket (OOP) payments, they can lead to poverty. The characteristics of the health system in Colombia imply that most of the costs of healthcare caused by smoking are absorbed by the national risk pool, leaving only a small portion of those costs to be payed by directly by the person/household (out-of pocket expenditure). Regarding the latter financial burden, it is estimated that the tax increase leads to a reduction of COP$194.2 MMM in OOP expenditure in healthcare (SDG-I3.8.2), representing protection from financial risk (SDG-T3.8).

#### Poverty (SDG1)

Households who are not poor can be pushed below the poverty line by high healthcare costs of NCDs treatment caused by smoking; thus, averted deaths from smoking translate into averted new cases of households falling into poverty. The estimates suggest that the tax hike averts 28.96 thousand people from falling into poverty. Along the same line, catastrophic expenditure on healthcare is defined as household’s out-of-pocket expenditure on healthcare higher than 10% of the household’s total income. The results suggest that the tobacco tax hike averts 337.6 thousand people from having catastrophic expenditure.

#### Income Equity (SDG10)

**FIGURE 3:**
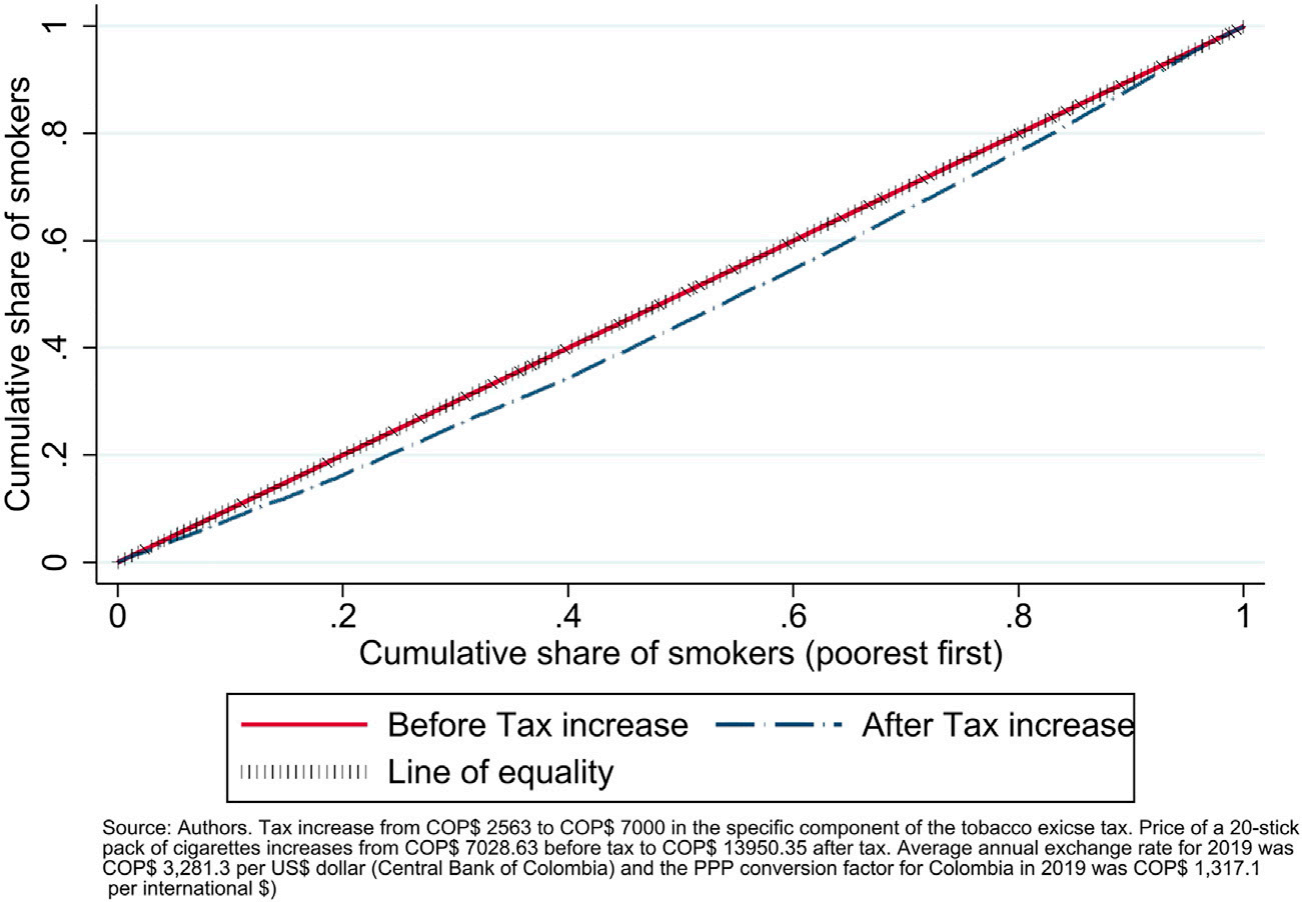
Concentration curve of smokers (Colombia, 2019).

Increases in tobacco taxes are progressive both in the short-run and in the long-run; in the short-run because individuals with lower income are more sensitive to changes in prices, and therefore the gains of changing from paying a positive amount of tax to paying zero tax because of quitting concentrate in those populations; in the long-run, the gains in health both in morbidity and mortality exceed by far, at the aggregate level, the additional costs imposed on smokers who did not quit [20, 39]. Figure 3 shows the Concentration Curve of smokers before and after the tax increase. Since current smokers are the focus of the study, before the tax increase the distribution perfectly matches the 45^*◦*^ line of equity. The dashed line shows that the tax increase benefits all segments of the population by reducing exposition to smoking all over the income distribution. However, the effect is not homogeneous; it can be seen that such benefit is higher in low-income individuals, showing that the distributional effect of the tax increase is progressive. This happens because the price elasticity is lower in low-income individuals, meaning that their quitting response to the tax increase is higher as compared to the middle or high income individuals, who also benefit from the policy but in a lower magnitude. In general, tobacco taxes act as a fiscal policy that contributes to greater equality (SDG-T10.4). When results are aggregated at the subnational level, the same mechanism leads to concentration of the gains of the tobacco tax increase in the poorest regions in the country. This subnational effect is particularly relevant for Colombia because subnational authorities are partially responsible for health insurance coverage of the population with the lowest income (subsidized regime), and regions with the highest needs are the ones with the lowest resources to invest in local public health actions to counteract and deal with the consequences of the tobacco epidemic. In addition, subnational authorities are in charge of tobacco tax administration.

#### Gender Equality (SDG5)

With gender being one of the defining characteristics of individuals in the artificial society, the microsimulation model allows to address, at least partially, some of the drivers of disadvantages in terms of poverty and health for women levered by a reduction in tobacco consumption, and it is an attempt to bring an intersectional approach to explore interactions and outcomes within households that are influenced by tobacco consumption [40]. The next lines describe the most general aspects of that analysis. Regarding the distribution of risk factor by sex, the estimated number of smokers by sex is 2.8 million men and 1.66 million women; the tobacco tax reduces this exposition to 2.17 million male smokers and 1.27 female smokers. One of the mechanisms included in the model is second-hand smoking, which is relevant to identify the consequences of the power relation between smoker members of the household and other members (mostly women and children) [41]. Since tobacco taxes reduce smoking rates, they contribute to disease prevention due to decreased exposure in the household [42]. We calculate that effect based on previous evidence on prevalence of second-hand smoking at home for Colombia [43], and on epidemiological parameters for morbidity and mortality from second-hand smoke [44, 45]. The results suggest that the second-hand smoke averted mortality is one of the effects of the policy affecting gender equity; despite the higher number and prevalence of male smokers, most of the averted deaths from second-hand smoke (8.71 M out of 15.9 M) correspond to women. In addition, there are other mechanisms operating in the model. First, the reduction of the crowding-out effect of tobacco expenditure caused by the tobacco tax increase studied in several LMIC, including Chile, with similar cultural and socioeconomic conditions [46]. Second, the averted loss in the pool of income at the household level. Both mechanisms suggest that the tobacco tax policy promotes gender equity.

### System 2: The Economy

#### Education

Human capital plays a central role on economic growth [47] as well as on development [48], with education and health [49] being “*the two most important sources of human capital: knowledge capital and health capital*” [50]. In addition to the loss in health capital represented by morbidity and mortality, smoking and NCDs lead to losses in knowledge capital *via* the years of education lost with premature death. For that reason, by averting deaths from smoking, tobacco taxes also contribute in avoiding losses in knowledge capital. The tax increase averts the loss of 10.1 years of education per smoker (≈ the number of years needed to complete middle school); adding up this effect for every quitter leads to a total of 3.37 MM of lost years of education averted by the policy, contributing to SDG-T4.4. This gain is a conservative estimate because, since the model is static, individuals do not increase their educational attainment over time; however, it is expected that younger cohorts increase their years of education over time and that structural changes in the educational system also lead to changes in population’s level of education.

#### Domestic Resources

Tobacco taxation is a source of tax revenues; therefore the tax increase opens fiscal space and strengthens domestic resource mobilization (SDG-T 17.1). The increase in the excise rate more than compensates for the previously mentioned drop in the number of cigarettes in the Colombian market, leading to an increase in tax revenue from COP$ 797.1 MMM to COP$ 1,359.3 MMM. There is an additional positive, although unfortunate, effect on tax revenues. Because tobacco taxes are low in Colombia, some cigarettes are introduced legally to the country, taxes are paid, and afterwards they are smuggled into other countries in the region. This market tactic has been reported in Ukraine [51] and Belgium [52].

When such mechanism is taken into account, the initial tax revenue from tobacco taxes is estimated at COP$1,089.7 MMM, and the tax hike increases it to COP$ 2,158.3 MMM. This is explained by the cigarette surplus entering Colombia: before the tax increase, it is estimated that 425.2 MM 20-stick packs of cigarettes enter legally into the country, and after the tax such quantity is expected to decrease to 308.3 MM packs. However, it is important to notice that the excess tax revenue is artificial in the sense that it is expected to significantly be reduced once Colombia catches up with the region in the level of tobacco taxes and effective policies to counter illicit cigarette trade [53] are accurately implemented [54], reducing corruption and organized crime, and strengthening rule of law (SDG16).

A final point of public finance is worth mentioning. The tax increase transfers resources from the tobacco industry to the government, and such transfer is socially desirable as the tobacco industry imposes health and environmental externalities for which the government, and the society in general, has to pay. Despite the tax increase, the results show that the profits from the tobacco industry are expected to increase from COP$ 236.8 MMM to COP$ 344.7 MMM. This unintended increase is caused by the tobacco industry’s typical response to tobacco taxes, overshifting the tax to the consumer to maintain and increase their profits [55]. The implication of this result is that even after an important increase in tobacco taxes as the one simulated in the model, there is still space for future further increases in tobacco taxes.

### The Earth’s System

The environmental footprint of tobacco is multidimensional [56]. The environmental component of the model focuses on post-consumption issues: incorrect disposal of cigarette butts and its related impact on water. Cigarette butts are the most common type of solid waste in urban and coastal areas [57, 58], they release harmful substances that contaminate water bodies, directly or indirectly [59]. To calculate the environmental effect, it has been established that out of 6 trillion cigarettes smoked globally, 4.5 trillion (75%) are littered in the environment [60]; in countries with lower waste disposal restrictions such as Colombia, it is plausible this ratio is even higher.

Since tobacco consumption is 332 million packs/year (i.e., 6.6 thousand million cigarettes), about 5 thousand million cigarette butts are littered in the environment. After the tax increase, the drop in consumption (2,307 million fewer cigarette sticks) would mean that 1,753 million cigarette butts were averted from ending in landfills (SDG-T12.5). Taking into account that one cigarette butt can pollute 1,000 L of water [61], the result suggests that the averted cigarette butts will avoid contamination of 1.7 trillion liters of water (SDG-T6.3).

## Discussion

The microsimulation model developed in this paper allows to evaluate the effects of tobacco taxes on SD by 1) keeping track of the dimensions of SD recorded in nationally representative surveys, ii) capturing the observed heterogeneity of the population and iii) extending and tailoring the model to accurately represent the particular conditions of the epidemic in a country. This overcomes usual limitations of macro-level analysis (e.g., [34]), mainly the drastic elimination of individual heterogeneity, which makes it unsuitable for a more nuanced SD approach in policy evaluation because such heterogeneity represents social types and it is fundamental for SD dimensions such as equity [4]. Compared to previous evaluations of tobacco taxation done for Colombia, the pricing model module and the inclusion of additional mechanisms such as second-hand smoke, provide methodological contributions to better tailor the model to the country’s conditions and offer a pathway for future assessments in countries with data availability.

Despite such contribution, one major limitation is that the model is static, that is, time is not explicitly modeled, and time periods, even when implicit, do not relate to each other. This has implications in the analysis. First, households in the model do not evolve over time, which is inconsistent with the observed natural recomposition of households over the life course. This aspect is relevant for SD dimensions such as gender equity and the role of women in the household, income over the life course, poverty traps and intergenerational poverty. Second, regarding smoking, the static nature of the model does not capture effects of age of initiation or individuals’ intention to start smoking.

Dynamic microsimulation [62] would be the obvious alternative because there is potential on explicitly incorporating dynamics in the model, and some dynamics are either theory or policy relevant. For tobacco control and SD, to our judgement, the most relevant dynamic mechanism is smoking behavior over the life course [63] in the five stages of addictive behavior [64]. Unfortunately, there is no data tracking smoking behavior over the life course for Colombia; besides suggesting to incorporate such dynamics in the model for future research, what is important for policy is the need for expanding data collection either from nationally representative surveys, cohort studies, or administrative records in Colombia to track individual smoking behavior over time.

In addition to incorporating dynamics at the micro level, there are other areas where the flexibility of the model can be exploited to extend the analysis for policy relevant discussions. One of such areas is the inclusion of vaping products and electronic cigarettes, because they also have effects on health and SD, and the emerging evidence of their potential as gateway for smoking tobacco [65]. Another area is price and income elasticity heterogeneity, mainly along the demand curve, which is especially relevant to accurately understand and evaluate the effect of big increases in excise taxes [20]. Finally, heterogeneity in prices coming from heterogeneity in products is another area that can potentially be accommodated in the model. This is an important element because different products target different segments of the population, and pricing strategies can vary widely across the spectrum of products, in order to keep the market share and also to position consumption in specific segments of the market.

Regardless of those potential methodological developments, the results of the analysis presented in this paper suggest that the societal impacts in SD from increases in tobacco taxes have been overlooked. A big increase in the specific component of the tobacco excise tax in Colombia remains as a key intervention for achieving considerable public health gains in Colombia. This study, in addition, quantifies how such increase is also a policy instrument to accelerate Colombia’s progress on multiple dimensions of SD.

## References

[1] Murray CJL Aravkin AY Zheng P Abbafati C Abbas KM Abbasi-Kangevari M Global burden of 87 Risk Factors in 204 Countries and Territories, 1990-2019: a Systematic Analysis for the Global Burden of Disease Study 2019. Lancet (2020) 396:1223–49. 10.1016/S0140-6736(20)30752-2 33069327 PMC7566194

[2] HHS. The Health Consequences of Smoking - 50 Years of Progress. A Report of the Surgeon General. Atlanta, GA: Department of Health and Human Services (HHS) (2014). Tech. rep. U.S.

[3] NCI and WHO. The Economics of Tobacco and Tobacco Control. Tech. Rep. NCI Tobacco Control Monograph Series 21. In: Department of Health and Human Services. Bethesda, MD; Geneva, CH: National Institutes of Health, U.S National Cancer Institute (NCI), World Health Organization (WHO) (2016).

[4] Rosa Dias P. Equality of Opportunity in Health. In: Culyer AJ, editor. Encyclopedia of Health Economics. Elsevier (2014). p. 282–6. ISBN: 978-0-12-375678-7. 10.1016/b978-0-12-375678-7.00210-8

[5] Solar O Irwin A. A Conceptual Framework for Action on the Social Determinants of Health. Geneva, CH: World Health Organization (WHO). (2010). Tech. rep. 2

[6] Samet JM Yoon S-Y, editors. Gender, Women and the Tobacco Epidemic. Geneva, CH: World Health Organization (WHO) (2010). ISBN: 9789241599511.

[7] FCTC. Gender-Responsive Tobacco Control: Evidence and Options for Policies and Programmes. Geneva, CH: World Health Organization (WHO) Framework Convention on Tobacco Control, Secretariat (2018). Tech. rep.

[8] Grown C Valodia I. Taxation and Gender Equity. A Comparative Analysis of Direct and Indirect Taxes in Developing and Developed Countries. New York, NY: Routledge (2010). ISBN: 978–0–415–56822–7.

[9] Schofield D Carter H Edwards K. Health Models. In: O’Donoghue CE, editor. Handbook of Microsimulation Modelling. London, United Kingdom: Emerald (2014). Chap. Ch.14. ISBN: 978-1-78350-570-8. 10.1108/S0573-8555201429310.1108/s0573-855520140000293013

[10] Pichon-Riviere A Bardach A Augustovski F Alcaraz A Reynales-Shigematsu LM Pinto MT Economic Impact of Smoking on Health Systems in Latin America: A Study of Seven Countries and its Extrapolation to the Regional Level. Pan Am J Public Health (2016) 40:1–9. 28001196

[11] Pichon-Riviere A Augustovski F Bardach A Colantonio L. Development and Validation of a Microsimulation Economic Model to Evaluate the Disease Burden Associated with Smoking and the Cost-Effectiveness of Tobacco Control Interventions in Latin America. Value in Health (2011) 14:S51–S59. 10.1016/j.jval.2011.05.010 21839900

[12] Yang W Zou Q Tan E Watkins L Beronja K Hogan PF Future Health and Economic Impact of Comprehensive Tobacco Control in DoD: A Microsimulation Approach. Mil Med (2018) 183:e104–e112. 10.1093/milmed/usx015 29401346

[13] Kim D Chen C Tysinger B Park S Chong MZ Wang L Smoking, Life Expectancy, and Chronic Disease in South Korea, Singapore, and the United States: A Microsimulation Model. Health Econ (2019) 30:92–104. 10.1002/hec.3978 31802569 PMC7269831

[14] IOM. In: Wallace R Geller A OgawaAyano V, editors. Assessing the Use of Agent-Based Models for Tobacco Regulationlish. Washington, DC: Institute of Medicine (IOM), The National Academies Press (2015). 10.17226/19018 26247084

[15] Sen A. Chapter 1 the Concept of Development. In: Chenery H Srinivasan TN, editors. Handbook of Development Economics, Vol. 1. Elsevier (1988). p. 9–26. Chap. Chapter 1, ISBN: 9780444703378. 10.1016/S1573-4471(88)01004-6

[16] UN. Agenda for Development. New York (NY): United Nations (1997). Tech. rep. A/RES/51/240. United Nations (UN), fifty-first session.

[17] UN. Transforming Our World: The 2030 Agenda for Sustainable Development. New York (NY): United Nations (2015). Tech. rep. A/70/L.1. United Nations (UN). General Assembly. Seventieth session.

[18] Sachs J. The Age of Sustainable Development. Columbia University Press (2015). ISBN: 978–1515910879.

[19] Chaloupka FJ Jeffrey D Erika S Violeta V Michal S Maryam M Tobacconomics Cigarette Tax Scorecard. 2nd ed. Chicago, IL: Health Policy Center, Institute for Health Research and Policy, University of Illinois Chicago (2021). Tech. rep.

[20] Marquez P Moreno-Dodson B. Tobacco Tax Reform at the Crossroads of Health and Development : A Multisectoral Perspective. Washington, DC: World Bank (2017). Tech. rep.

[21] UNDP. Investment Case for Tobacco Control in Colombia. In: The Case for Scaling-Up WHO FCTC Implementation. New York (NY): United Nations (2019). Tech. rep. United Nations Development Program (UNDP), Who Framework Convention on Tobacco Control Secretariat (FCTC), Pan American Health Organization (PAHO), Research Triangle Institute (RTI).

[22] Minsalud and OPS. Impuestos al tabaco en Colombia. Actualización de cifras en el marco del proyecto Framework Convention for Tobacco Control 2030. Washington (DC): Organización Panamericana de la Salud (2021). Tech. rep. Ministerio de Salud y Protección Social, Republica de Colombia (Minsalud), Organización Panamericana de la Salud (OPS).

[23] Congreso de la República de Colombia. Proyecto de Ley 365 de 2020C. Bogotá, Colombia: Congreso de la República (2020).

[24] Jha P Marquez P Dutta S. Tripling Tobacco Taxes: Key for Achieving the UN Sustainable Development Goals by 2030. Washington (DC): Published in Investing in Health: World Bank Blogs, world Bank (2017).

[25] Richiardi M. Artificial Economics and Self Organization. In: Leitner S Wall F, editors. Artificial Economics and Self Organization: Agent-Based Approaches to Economics and Social Systems. Springer International Publishing (2014). ISBN: 978-3-319-00912-4. 10.1007/978-3-319-00912-4

[26] DANE. Metodología General Encuesta Nacional de Calidad de Vida. República de Colombia: Producción Estadística PES, Dirección de Metodología y Producción Estadística DIMPE, Departamento Administrativo Nacional de Estadística (DANE) (2020). Tech. rep.

[27] Krumpal I. Determinants of Social Desirability Bias in Sensitive Surveys: a Literature Review. Qual Quant (2013) 47:2025–47. 10.1007/s11135-011-9640-9

[28] DANE and Minjusticia. Metodología General. Encuesta Nacional de Consumo de Sustancias Psicoactivas en población general (ENCSPA). República de Colombia: Producción Estadística - PES, Dirección de Metodología y Producción Estadística - DIMPE, Departamento Administrativo Nacional de Estadística (DANE) y Ministerio de Justicia y del Derecho (Minjusticia) (2020). Tech. rep.

[29] OID. Inter-American Uniform Drug Use Data System. Protocol of the Household Survey on Drug Use. Instruments to Conduct National Studies on Drug Use in the General Population. Washington (DC): Organization of American States (2011). Tech. rep. Inter-American Observatory on Drugs (OID), Comisión Interamericana para el control del abuso de drogas (CICAD), Organization of American States (OAS).

[30] Thomopoulos NT. Essentials of Monte Carlo Simulation. Statistical Methods for Building Simulation Models. Springer (2013). ISBN: 978-1-4614-6022-0. 10.1007/978-1-4614-6022-0.

[31] DANE. Encuesta Nacional de Calidad de Vida 2018. In: Metodología de cálculo de la variable ingreso. República de Colombia: Departamento Administrativo Nacional de Estadística (DANE) (2019). Tech. rep.

[32] Verguet S Kim JJ Jamison DT. Extended Cost-Effectiveness Analysis for Health Policy Assessment: A Tutorial. In: , 34 (2016). p. 913–23. 10.1007/s40273-016-0414-z PharmacoEconomics 27374172 PMC4980400

[33] Verguet S Jamison DT. Health Policy Analysis: Applications of Extended Cost-Effectiveness Analysis Methodology. In: Jamison DT, editor. Disease Control Priorities: Improving Health and Reducing Poverty. 3rd ed., Vol. 9. Washington, DC: The International Bank for Reconstruction and Development/The World Bank (2017). Chap. Ch.8. ISBN: 978-1-4648-0527-1. 30212165

[34] Global Tobacco Economics Consortium. The Health, Poverty, and Financial Consequences of a Cigarette price Increase Among 500 Million Male Smokers in 13 Middle Income Countries: Compartmental Model Study. BMJ (2018) 361:k1162. 10.1136/bmj.k1162 29643096 PMC5894369

[35] Nugent R Bertram MY Jan S Niessen LW Sassi F Jamison DT Investing in Non-communicable Disease Prevention and Management to advance the Sustainable Development Goals. The Lancet (2018) 391:2029–35. 10.1016/S0140-6736(18)30667-610.1016/s0140-6736(18)30667-6 29627167

[36] WHO. WHO Framework Convention on Tobacco Control. Geneva, CH: World Health Organization (WHO) (2004). Tech. rep.

[37] Chang JT Anic GM Rostron BL Tanwar M Chang CM. Cigarette Smoking Reduction and Health Risks: A Systematic Review and Meta-Analysis. Nicotine Tob Res (2021) 23:635–42. 10.1093/ntr/ntaa156 32803250

[38] CNMH. Basta Ya! Colombia: Memorias de guerra y dignidad. Informe general, Grupo de Memoria Histórica. In: Centro Nacional de Memoria Histórica (CNMH) (2013). ISBN: 978-958-57608-4-4.

[39] Fuchs A Icaza G Fernanda PD. Distributional Effects of Tobacco Taxation : A Comparative Analysis. Tech. Rep. In: Policy Research Working Paper;No. 8805. Washington, DC: World Bank (2019).

[40] Kapilashrami A Hankivsky O. Intersectionality and Why it Matters to Global Health. The Lancet (2018) 391:2589–91. 10.1016/S0140-6736(18)31431-410.1016/s0140-6736(18)31431-4 30070211

[41] George AS Amin A GarcÍa-Moreno C Sen G. Gender equality and Health: Laying the Foundations for Change. The Lancet (2019) 393:2369–71. 10.1016/S0140-6736(19)30987-010.1016/s0140-6736(19)30987-0 31155277

[42] Vavalà T Rigney M Reale ML Novello S King JC. An Examination of Two Dichotomies: Women with Lung Cancer and Living with Lung Cancer as a Chronic Disease. Respirology (2020) 25:24–36. 10.1111/resp.13965 33124087

[43] Fundacion Salutia. ERICA: Encuesta de Riesgo Cardiovascular 2017 (2018). ISBN: 978-958-58266-7-0.

[44] Yousuf H Hofstra M Tijssen J Leenen B Lindemans JW van Rossum A Estimated Worldwide Mortality Attributed to Secondhand Tobacco Smoke Exposure, 1990-2016. JAMA Netw Open (2020) 3:e201177. 10.1001/jamanetworkopen.2020.1177 32181828 PMC7078760

[45] Max W Sung H-Y Shi Y. Deaths from Secondhand Smoke Exposure in the United States: Economic Implications. Am J Public Health (2012) 102:2173–80. 10.2105/AJPH.2012.300805) 22994180 PMC3477960

[46] Paraje G Araya D. Relationship between Smoking and Health and Education Spending in Chile. Tob Control (2018) 27:560–7. 10.1136/tobaccocontrol-2017-05385710.1136/tobaccocontrol-2017-053857 28986435 PMC6109233

[47] Savvides A Stengos T. Human Capital and Economic Growth. Stanford University Press (2009). ISBN: 978-0-8047-5540-5.

[48] Bleakley H. Health, Human Capital, and Development. Annu Rev Econ (2010) 2:283–310. 10.1146/annurev.economics.102308.124436 PMC3800109 24147187

[49] Becker GS. Health as Human Capital: Synthesis and Extensions. Oxford Econ Pap (2007) 59:379–410. 10.1093/oep/gpm020

[50] Grossman M. Chapter 10 Education and Nonmarket Outcomes. Handbook Econ Educ (2006) 1:577–633. 10.1016/S1574-0692(06)01010-510.1016/s1574-0692(06)01010-5–

[51] Lavrov V. Ukraine’s ‘lost’ Cigaretttes Flood Europe: Big Tobacco’s Overproduction Fuels $2 Billion Black Market. International Consortium of Investigative Journalists (2014).

[52] Chambers M. British-American Tobacco Ltd V HMRC First-Tier Tribunal (Tax Chamber). London, United Kingdom: Monckton Chambers (2017). 14 February 2017; [2017] UKFTT 167 (TC): Tribunal clarifies tobacco manufacturers’ anti-smuggling *duties* .

[53] WHO. WHO Protocol to Eliminate Illicit Trade in Tobacco Products (2013). Tech. rep. 23741895

[54] Llorente B Maldonado N. Colombia: Illicit Cigarette Trade. In: Dutta S, editor. Confronting Illicit Tobacco Trade: A Global Review of Country Experiences. Washington, DC: The World Bank (2019). p. 292–321.

[55] Gilmore AB Fooks G Drope J Bialous SA Jackson RR. Exposing and Addressing Tobacco Industry Conduct in Low-Income and Middle-Income Countries. Lancet (2015) 385(15):1029–43. 10.1016/S0140-6736(15)60312-9 25784350 PMC4382920

[56] Zafeiridou M Hopkinson NS Voulvoulis N. Cigarette Smoking: An Assessment of Tobacco's Global Environmental Footprint across its Entire Supply Chain. Environ Sci Technol (2018) 52:8087–94. 10.1021/acs.est.8b01533 29968460

[57] Araújo MCB Costa MF. A Critical Review of the Issue of Cigarette Butt Pollution in Coastal Environments. Environ Res (2019) 172:137–49. 10.1016/j.envres.2019.02.005 30782533

[58] WHO. Tobacco and its Environmental Impact: An Overview. Geneva, CH: World Health Organization (WHO) (2017). Tech. rep.

[59] Dobaradaran S Soleimani F Akhbarizadeh R Schmidt TC Marzban M BasirianJahromi R. Environmental Fate of Cigarette Butts and Their Toxicity in Aquatic Organisms: A Comprehensive Systematic Review. Environ Res (2021) 195:110881. 10.1016/j.envres.2021.110881 33607099

[60] Curtis C Novotny TE Lee K Freiberg M McLaughlin I. Tobacco Industry Responsibility for Butts: a Model Tobacco Waste Act. Tob Control (2017) 26:113–7. 10.1136/tobaccocontrol-2015-052737 26931480 PMC5256370

[61] Torkashvand J Farzadkia M Sobhi HR Esrafili A. Littered Cigarette Butt as a Well-Known Hazardous Waste: A Comprehensive Systematic Review. J Hazard Mater (2020) 383:121242. 10.1016/j.jhazmat.2019.121242 31563043

[62] Li J 0’Donoghue C Dekkers G. Dynamic Models. In: 0’Donoghue CE, editor. Handbook of Microsimulation Modelling (2014). Chap. Ch.10. ISBN: 978-1-78350-570-8. 10.1108/S0573-8555201429310.1108/s0573-855520140000293009

[63] Lillard D Christopoulou R. Life-Course Smoking Behavior. Oxford University Press (2015). ISBN: 978–0–19–938910–0.

[64] Prochaska JO DiClemente CC Norcross JC. In Search of How People Change: Applications to Addictive Behaviors. Am Psychol (1992) 47:1102–14. 10.1037//0003-066x.47.9.110210.1037/0003-066x.47.9.1102 1329589

[65] Chapman S Bareham D Maziak W. The Gateway Effect of E-Cigarettes: Reflections on Main Criticisms. Nicotine Tob Res (2019) 21:695–8. 10.1093/ntr/nty067 29660054 PMC6468127

[66] BAT. Performance Summary 2019. Cautionary Statement and Other Information. British American Tobacco (BAT). p. 2020. Tech. rep. p.l.c. (No. 3407696).

[67] Branston R. Addressing Industry Profits as Part of Tobacco Control. Union for International Cancer Control (2021).

[68] Maldonado N Llorente B Escobar D Iglesias RM. Smoke Signals: Monitoring Illicit Cigarettes and Smoking Behaviour in Colombia to Support Tobacco Taxes. Tob Control, 29 (2019). p. s243–s248. Published.04 May 2019. 10.1136/tobaccocontrol-2018-054820 31055351

[69] Meléndez M Vásquez T. Análisis de la competencia en la industria colombiana de cigarrillos. Bogota, Colombia: Fedesarrollo (2009).

[70] Congreso de la República de Colombia. Proyecto de Ley 339 de 2020C. Bogota, Colombia: Congreso de la Republica (2020).

[71] Congreso de la República de Colombia. Proyecto de Ley 010 de 2020S. Bogota, Colombia: Congreso de la Republica (2020).

[72] Peto R. Smoking and Death: the Past 40 Years and the Next 40. Bmj (1994) 309:937–9. 10.1136/bmj.309.6959.937 7950669 PMC2541135

[73] Lam TH Ho SY. Tobacco. In: Detels R, editor. Oxford Textbook of Global Public Health. 6th ed. Oxford University Press (2015). p. 1217–32. Chap. 9.1. ISBN: 9780199661756. 10.1093/med/9780199661756.003.0219

[74] Camacho S Maldonado N Bustamante J Llorente B Cueto E Cardona F How Much for a Broken Heart? Costs of Cardiovascular Disease in Colombia Using a Person-Based Approach. PLOS ONE (2018) 13:e0208513. 10.1371/journal.pone.0208513 30566516 PMC6300287

[75] DANE. Actualización de las líneas de pobreza monetaria. República de Colombia: Departamento Administrativo Nacional de Estadística (DANE) (2020). Tech. rep.

[76] DANE. Pobreza monetaria en Colombia. República de Colombia: Departamento Administrativo Nacional de Estadística (DANE) (2018). Tech. rep. Boletín técnico COM-030-PD-001-r-004 V8.

[77] Smith KE Savell E Gilmore AB. What Is Known about Tobacco Industry Efforts to Influence Tobacco Tax? A Systematic Review of Empirical Studies. Tob Control (2013) 22:144–53. 10.1136/tobaccocontrol-2011-05009810.1136/tobaccocontrol-2011-050098 22887175 PMC3701860

[78] Bank W. Confronting Illicit Tobacco Trade: A Global Review of Country Experiences, editor. Dutta S, Washington, DC: The World Bank (2019).

[79] Ross H. Understanding and Measuring Cigarette Tax Avoidance and Tax Evasion. A Methodological Guide. Tech. Rep. Prepared for the Economics of Tobacco Control Project, School of Economics. Chicago, IL: University of Cape Town, Tobacconomics, Health Policy Center, Institute for Health Research, and Policy, University of Illinois at Chicago. (2015).

[80] Maldonado N Llorente BA Iglesias RM Escobar D. Measuring Illicit Cigarette Trade in Colombia. Tob Control (2018) 29:s260–s266. 10.1136/tobaccocontrol-2017-053980 29540558

[81] Gallego JM Llorente B Maldonado N Otálvaro-Ramírez S Rodríguez-Lesmes P. Tobacco Taxes and Illicit Cigarette Trade in Colombia. Econ Hum Biol (2020) 39:100902. 10.1016/j.ehb.2020.100902 32622932

[82] van Walbeek C. Measuring Changes in the Illicit Cigarette Market Using Government Revenue Data: the Example of South Africa. Tob Control 23 (2014), pp. e69–e74. 10.1136/tobaccocontrol-2013-051178 24431121 PMC4078715

[83] Stoklosa M. Is the Illicit Cigarette Market Really Growing? the Tobacco Industry's Misleading Math Trick: Table 1. Tob Control (2016) 25:360–1. 10.1136/tobaccocontrol-2015-052398 26001698

