# Supplementary material for: Tobacco Taxes as the Unsung Hero: Impact of a Tax Increase on Advancing Sustainable Development in Colombia
Source: Int J Public Health. 2022 Mar 30;67:1604353. doi: 10.3389/ijph.2022.1604353 (PMC9006985; doi:10.3389/ijph.2022.1604353)
Supplement: Supplementary file 1 [file DataSheet1.pdf]

C Supplementary file 1: Model for simulation of behaviors and states

C.1 Tax and price of cigarettes: Baseline Scenario

This block has three consecutive components, namely, taxes, exwork prices before taxes, and retail prices.

Tax structure. The starting point of the model is the increase in the excise tax for cigarettes. According to the current tax legislation in Colombia, the tax on cigarettes has three components: (i) the general VAT with a rate of 19% of the exwork price, (ii) an ad-valorem excise tax with a rate of 10% of the of the previous year average price by brand measured by an official survey in supermarkets performed annually by the National Department of Statistics, and (iii) a specific excise tax of COP$2,563 per pack of 20 cigarettes (henceforth pack). Since exwork prices are not observed, we calculate the implicit exwork price based on the tax rates and tax value reported in the receipt of a pack bought in a supermarket. The observed baseline tax value for VAT is COP$524.13 suggesting an implicit exwork price before VAT of COP$2,758.6 (524/0.19); meanwhile, the median of the observed price in DANE's supermarkets survey (COP$ 6,069.1 per pack) was used to calculate the advalorem excise tax at COP$ 606.91. For the specific component, the current rate is COP$ 2,563. Adding the value of all three taxes leads to a total tax of COP$ 3,694.

Exwork pricing The departing point is the manufacturing cost of a 20-cigarette pack, which has been estimated at COP$ 1,000 [84].We add a minimum profit rate of 20%, a modest assumption, considering that the reported operating margins by the two main multinational companies that supply the Colombian cigarette market are above 27% [66, 67]. This profit rate translates in a minimum profit of COP$ 200 per pack. In addition to the minimum profit rate, tobacco companies also benefit from the pass/through, that is, from overshifting or undershifting prices [55]; for Colombia, the evidence [68, p.s247] suggests overshifting behavior of 74% (60.9/35). In order to be conservative in the simulation of the policy, we use a value of 20% for the overshifting parameter, which in turn leads to a profit from overshifting of COP$ 512.6 per pack. Adding both profits and the manufacturing cost leads to a exwork price before taxes of COP$ 1,712.6.

Retail price. To get to the retail price, we add a profit margin for distribution of 15% over the exwork price after taxes, and another 15% for retail. This value informed by two sources. The information gathered from one focus group in a Colombian cigarette market concentration study [69], and the price charts distributed by tobacco companies to retailers. Since the exwork price is COP$ 1,712.6 and the total value of tax is COP$ 3,694, the exwork price after taxes is COP$ 5,406.6; adding the 30% of distribution and retail margins leads to a retail price for consumers of COP$ 7,028.6, which is consistent the the observed average price of a pack in Colombia.

C.2 Tax and price of cigaretes: Policy Scenario

The policy simulated with the model is an increase of the specific component of the excise tax from COP$ 2,563 to COP$ 7,000 per pack; all equations and all the other parameters of the pricing model remain the same.

In this scenario, the VAT and advalorem component of the excise remain the same, and the total value of taxes per pack becomes COP$ 8,131 (524.1 + 606.9 + 7,000). The manufacturing profit goes up from COP$712.6 per pack to COP$ 1,600 per pack, due to the overshifting behavior of the tobacco industry, leading to an exwork price before taxes of COP$ 2,600. Adding this price with the value of taxes leads to an exwork price after taxes of COP$ 10,731 (2,600 + 8,131), and applying the distribution and retail margins leads to an expected retail price after the tax of COP$ 13,950.4. For robustness and to provide additional input for policy dialogue, we added a sensitivity analysis by simulating two alternative policy scenarios. The first one is a modest increase of the specific component to COP$2,600, based on the congressional initiative 339/2020 [70, Article 7]. The second one, is a moderate increase to COP$4,200, based on the congressional initiative 010/2020 [71, Article 59]. All the three scenarios considered in the analysis match the proposals in three different bills presented to the Colombian Congress during 2019-2020 [22]; thus, the values are relevant for decision making, notwithstanding the possibility of using the model to simulate other values.

The final component of the economic block of the model is the economic behavior of smokers, that is, the behavioral response to increases in prices caused by the tax change. We use the same values of price elasticities by income quintile presented in [34] for Colombia, that is, a price elasticity of -0.635 for the lowest income quintiles, -0.122 for the highest income quintile, and a linear interpolation of those values for each of the remaining three quintiles. Also, following [34], the model assumes that half of the impact of elasticity goes to reduction at the extensive margin, that is, to smokers quitting, and half goes to a reduction at the intensive margin, that is, reduction in the number of cigarettes smoked in the non-quitting smokers.

The tax and pricing module is the starting point of the model. Formally, it gives a change in price $\Delta p = 0.985$, a decline in the number of smokers (extensive margin) of $\Delta SmokingExt_y = 0.5\epsilon_y$, where ϵ_y is the cigarette price elasticity for smokers in income quintile $y = 1, \ldots, 5$, and a reduction in smoking intensity (intensive margin) of the non/quitting smokers of $\Delta SmokingInt_y = (1 - 0.5)\epsilon_y$.

C.3 Smoking

Smoking changes at the extensive level based on the number of smokers in the baseline scenario $Smokers_{0,y}$ (available at the micro level in the synthetic dataset), and the change in this number caused by the tax increase $\Delta SmokingExt_y$. Formally, the number of smokers who quit after the tax increase $SQuit_T$ is

$$SQuit_{T,y} = Smokers_{0,y} \times \Delta SmokingExt_y \tag{1}$$

This is operationalized at the micro level in the microsimulation by a random draw of $SQuit_{T,y}$ smokers from the synthetic dataset using a uniform distribution, and changing the status of the individuals randomly selected from smokers to quitters. All random draws in the microsimulation

are done using a uniform distribution; to avoid redundancy, from now on we omit mentioning this detail.

The number of cigarettes falls in part because consumption of quitters drops to zero A second effect bringing down the number of cigarettes consumed comes from smokers who did not quit but reduce their smoking intensity. Formally, the number of cigarettes smoked by a non-quitter i in income quintile y after the tax increase, $SCiga^{i}_{y,T}$, is

$$SCiga^{i}_{y,T} = SCiga^{i}_{y,0} \times \Delta SmokingInt_{y} \tag{2}$$

C.4 Health

Deaths before the tax increase

Based on the epidemiological evidence [72, 73], it is expected that half of smokers in the baseline scenario will die from smoking. Formally

$$HDeaths_{0} = 0.5 \sum_{y} Smokers_{0,y} \tag{3}$$

In the microsimulation, $HDeaths_{0}$ pre-tax smokers are randomly selected and marked as expected to die from smoking. For Second-Hand Smoking (SHS), we concentrate on SHS at home, based on previous evidence for Colombia showing that this is the most important source of SHS [43], as a consequence of a comprehensive smoke-free area regulation in public spaces. We identify households in the artificial society with at least one smoker and one non-smoker, and then we calculate the number of deaths due to SHS by dividing the total number of smokers living in those households by 56.1, the parameter estimated by [44, Table 2] for Latin America and the Caribbean representing the *"number of individuals who smoked associated with the death of 1 individual who did not'"*. The estimated number of deaths from SHS before the tax increase translates in a random draw from the population affected, namely, non-smokers living in the same household with smokers.

Deaths after the tax increase

After the tax increase, the number of deaths from smoking is reduced mainly from the risk reduction on quitters and from the reduction of SHS. The risk reduction is the proportion of smoking death risk reduced by quitting at an early age; the values by group of age, taken from [34], are shown in Table 4.

In the microsimulation, the risk reduction by age group is allocated to smokers who quit; then, those smokers are sorted randomly, their count accumulates over the population, and death is allocated to those who fall bellow the threshold of risk reduction. Formally

$$DeathQuitter_{j} = \begin{cases} 1 & \text{if } \sum_{k=1}^{j} w_{k} / \sum_{k=1}^{Q} w_{k} < RiskReduction \\ 0 & \text{Otherwise} \end{cases} \tag{4}$$

Table 4: Risk reduction from quitting smoking (Colombia, 2019 [34])

Age group	Risk reduction
[0,14]	1
[15,19]	0.9688
[20,24]	0.9477
[25,29]	0.9210
[30,34]	0.8925
[35,39]	0.8656
[40,44]	0.8368
[45,49]	0.7950
[50,54]	0.7290
[55,59]	0.6283
[60,64]	0.4992
[65,69]	0.3644
[70,74]	0.2469
[75,79]	0.1568
[80,84]	0.0908
[85,89]	0.0452
[90,94]	0.0163
[95,104]	0.0004

where j is the quitter, k is a counter for quitters, Q is the total number of quitters, and w_k is the frequency weight in the synthetic dataset for quitter k (i.e. the number of people this individual represents in the survey).

For SHS, non-smokers who live in the same household of a quitter reduce the exposition to SHS to zero. Therefore, all individuals exposed to SHS who were randomly assigned to die due to SHS and who live in the same household of a quitter change their probability of death from SHS from 1 to zero.

Life Years gained

The gains in life years are calculated based on the data provided in [34], shown in Table 5.

Table 5: Life years gained from quitting smoking (Colombia, 2019 [34])

Age group	Risk reduction
0	0.00
15	10.00
25	9.00
45	6.00
65	3.00
105	0.00

We do a cubic spline to interpolate those values for every year of age, and match it with the quitter's

age.

C.4.1 Non-Communicable Diseases

The deaths caused by smoking are allocated to the four main Non-Communicable Diseases related to tobacco consumption, namely, cardiovascular disease, stroke, Chronic Obstructive Pulmonary Disease (COPD) and Lung Cancer. The values to allocate those deaths among these four diseases are the ones used for Colombia in [34], and are shown in Table 6. For deaths caused by SHS, the data

Table 6: Share of NCDs on deaths caused by smoking (Colombia, 2019 [34])

NCD	Share (%)
Stroke	22.93
Cardiovascular Disease	52.33
COPD	18.50
Lung Cancer	6.76

comes from [45], and it is shown in Table 7.

Table 7: Share of NCDs on deaths caused by SHS (Colombia, 2019 [34])

NCD	Share (%)
Stroke	10.81
Cardiovascular Disease	39.57
COPD	41.27
Lung Cancer	8.33

Both the deaths from smoking and the ones from SHS are randomly allocated among the individuals whose death is caused by smoking and is averted by the tax increase.

C.5 Healthcare

Utilization of healthcare followed the modeling and parameters presented in [34] for Colombia. In particular, we use the gradient of utilization of healthcare representing differences in access to healthcare among income quintiles, as shown in Table 9.

Table 8: Gradient of healthcare utilization by income quintile (Colombia, 2019 [34])

Income Quintile	Gradient
1	1.002717
2	1.092391
3	1.000000
4	1.058424
5	1.173913

The probability of using healthcare is 0.7 [34], and the gradient in Table 9 combined with such probability gives the probability of effective use of healthcare for a person of a certain income quintile.

The per-capita cost of using healthcare is calculated for each NCD as the annual cost of a person with the disease in Colombia's health system applying the person-based approach [74] to the administrative records of healthcare utilization and costs in the contributory regime in Colombia (known as *"Base de suficiencia"*). The per capita costs in 2018 Colombian Pesos (COP$) are shown in Table 9.

Table 9: Healthcare costs (Colombia, 2018)

NCD	Annual cost per person (2018 COP$)
COPD	5,744,358
Stroke	9,548,821
Cardiovascular disease	4,703,103
Cancer	14,665,237

Two additional parameters are needed for the healthcare module, and both are taken from [34]. The first one is the portion of a healthcare bill that will be reimbursed to the person, or the proportion of the bill that is covered by health insurance; For Colombia this value is 1. The second parameter is the proportion of people covered by social health insurance, which is 91% for Colombia.

C.6 Poverty

The poverty line is estimated in Colombia by DANE using the basket of goods and services defined in the National Survey of Income and Expenditures (ENPH) and multiplying this value by the Orshansky coefficient [75]. The value used in the model was Colombia's 2018 poverty line of $ 257,433 Colombian Pesos (COP$) and the extreme poverty line of COP$ 117,605 [76].

There are two measures of poverty in the model. The first one is the proportion of households pushed into poverty due to out-of-pocket expenditures on healthcare, and consists of comparing the household percapita income minus the out-of-pocket expenditure in healthcare with the poverty line. The second one is catastrophic expenditure on healthcare, defined by the Worldbank as out-of-pocket healthcare expenditure representing more than 10% of household income.

C.7 Other elements

The model has some additional parameters used for either the modeling structure or as inputs for estimations of outputs. One of the additional elements is illicit cigarette trade, which has been a frequent argument of the tobacco industry to deter governments from increasing tobacco taxes [77], [20, Ch.9]. There is significant work on analyzing the nature, causes, consequences and effective interventions on illicit trade [53, 78, 79], and scientific evidence about this issue in Colombia has been building over time [80, 68, 54, 81]. For that reason, we included a module on illicit trade that works as follows. We take the total number of cigarettes entering the market from DANE's records of imports. Since domestic production is negligible since 2019, it is reasonable to assume total imports represent the legal cigarettes entering to Colombia, either to be consumed in Colombia

or to be reexported through licit or illicit channels to other countries in the region. Since the specific component of the tax is collected when cigarettes are imported, tax revenues before the tax increase are calculated based on imports, and tax revenues after the tax are calculated as the product between the new specific tax and the imports net of the drop in national consumption. The size of the illicit trade, based on the evidence for Colombia, is 6.4% of the cigarettes consumed. Therefore, we estimate the size of illicit trade applying this parameter to the total consumption of cigarettes obtained from the microsimulation. After the tax increase, we assume the level of illicit trade remains constant, because the evidence suggests the industry is not expected to directly or indirectly increase the amount of illicit cigarettes in response to the tax [82]. Also, literature reviewed in [3] indicates that large-scale cigarette illicit trade (such as the one more common in Colombia [54]) is largely determined by governance conditions: no changes in such conditions are expected, and thus it is reasonable to assume a stable size (in volume, not as market share). Since the level of illicit trade is constant and the national market shrinks due to the tax, the share of illicit cigarettes as proportion of cigarettes consumed may grow [83].

References

[66] BAT. *Performance Summary 2019: Cautionary statement and other information*. Tech. rep. British American Tobacco (BAT) p.l.c. (No. 3407696), 2020.

[67] Branston, Rob. *Addressing industry profits as part of tobacco control*. 2021.

[68] Maldonado, Norman et al. "Smoke signals: monitoring illicit cigarettes and smoking behaviour in Colombia to support tobacco taxes". In: *Tobacco control* Published.04 May 2019 (2019), pp. 1–6. DOI: 10.1136/tobaccocontrol-2018-054820.

[69] Meléndez, Marcela and Vásquez, Tatiana. "Análisis de la competencia en la industria colombiana de cigarrillos". 2009.

[70] Congreso de la República de Colombia. *Proyecto de Ley 339 de 2020C*. 2020.

[71] Congreso de la República de Colombia. *Proyecto de Ley 010 de 2020S*. 2020.

[72] Peto, R. "Smoking and death: the past 40 years and the next 40". eng. In: *BMJ (Clinical research ed.)* 309.6959 (Oct. 1994), pp. 937–939. DOI: 10.1136/bmj.309.6959.937.

[73] Lam, Tai -Hing and Ho, Sai -Yin. "Tobacco". In: *Oxford Textbook of Global Public Health*. Ed. by Detels, Roger et al. 6th Ed. Oxford University Press, 2015. Chap. 9.1. ISBN: 9780199661756. DOI: 10.1093/med/9780199661756.003.0219.

[74] Camacho, Sandra et al. "How much for a broken heart? Costs of cardiovascular disease in Colombia using a person-based approach". In: *PLOS ONE* 13.12 (2018), e0208513.

[75] DANE. *Actualización de las líneas de pobreza monetaria*. Tech. rep. Departamento Administrativo Nacional de Estadística (DANE), República de Colombia, 2020.

[76] DANE. *Pobreza monetaria en Colombia*. Tech. rep. Boletín técnico COM-030-PD-001-r-004 V8. Departamento Administrativo Nacional de Estadística (DANE), República de Colombia, 2018.

[77] Smith, Katherine E, Savell, Emily, and Gilmore, Anna B. “What is known about tobacco industry efforts to influence tobacco tax? A systematic review of empirical studies”. In: *Tobacco Control* 22.2 (Mar. 2013), e1 LP –e1. DOI: 10.1136/tobaccocontrol-2011-050098.

[78] World Bank. *Confronting Illicit Tobacco Trade: A Global Review of Country Experiences*. Ed. by Dutta, Sheila. The World Bank, 2019.

[79] Ross, Hana. *Understanding and measuring cigarette tax avoidance and tax evasion. A methodological guide*. Tech. rep. Prepared for the Economics of Tobacco Control Project¸ School of Economics, University of Cape Town, Tobacconomics, Health Policy Center, Institute for Health Research, and Policy, University of Illinois at Chicago., 2015.

[80] Maldonado, Norman et al. “Measuring illicit cigarette trade in Colombia”. In: *Tobacco control* (2018), pp. 2017–053980. DOI: 10.1136/tobaccocontrol-2017-053980.

[81] Gallego, Juan M et al. “Tobacco taxes and illicit cigarette trade in Colombia”. In: *Economics & Human Biology* 39 (2020), p. 100902. DOI: 10.1016/j.ehb.2020.100902.

[82] Walbeek, Corné van. “Measuring changes in the illicit cigarette market using government revenue data: the example of South Africa.” eng. In: *Tobacco control* 23.e1 (May 2014), pp. 69–74. DOI: 10.1136/tobaccocontrol-2013-051178.

[83] Stoklosa, Michal. “Is the illicit cigarette market really growing? The tobacco industry's misleading math trick”. In: *Tobacco Control* 25.3 (2016), pp. 360–361. DOI: 10.1136/tobaccocontrol-2015-052398

[84] Joossens, Luk, Raw, Martin. “From cigarette smuggling to illicit tobacco trade”. In: Tobacco Control 21 (2012), pp. 230-234. DOI: 10.1136/tobaccocontrol-2011-050205
